# ALA and ALA hexyl ester-induced porphyrin synthesis in chemically induced skin tumours: the role of different vehicles on improving photosensitization

**DOI:** 10.1054/bjoc.2001.2156

**Published:** 2001-11

**Authors:** A Casas, C Perotti, H Fukuda, L Rogers, A R Butler, A Batlle

**Affiliations:** Centro de Investigaciones sobre Porfirinas y Porfirias (CIPYP), CONICET and Department of Biochemistry, School of Sciences, University of Buenos Aires, Argentina; School of Chemistry, University of St Andrews, St Andrews, Fife, UK

**Keywords:** ALA, ALA esters, photodynamic therapy, squamous papillomas

## Abstract

Exogenous administration of 5-aminolevulinic acid (ALA) is becoming widely used to enhance the endogenous synthesis of Protoporphyrin IX (PpIX) in photodynamic therapy. We analysed porphyrin formation in chemically induced squamous papillomas, after topical application of ALA and ALA hexyl ester (He-ALA) administered in different formulations, as well as the pattern of distribution in the internal organs, and the synthesis of porphyrins in distant tumoural and normal skins. A lotion formulation containing DMSO and ethanol was the best vehicle for topical ALA delivery to papillomas, whereas cream was the most efficient formulation for He-ALA application. Similar porphyrin concentration can be accumulated in the skin tumours employing either ALA or He-ALA delivered in their optimal formulations. The use of cream as a vehicle of both ALA and He-ALA, induces highest porphyrin tumour/normal skin ratios. The main advantage of using He-ALA is that porphyrins synthesized from the ester are more confined to the site of application, thus inducing low porphyrin levels in normal skin, liver, blood and spleen, as well as in papillomas distant from the point of application, independently on the vehicle employed, so reducing potential side effects of photodynamic therapy. © 2001 Cancer Research Campaign http://www.bjcancer.com


*British Journal of Cancer* (2001) **85**(11), 1794–1800
© 2001 Cancer Research Campaign
doi: 10.1054/ bjoc.2001.2156, available online at http://www.idealibrary.com on IDEAL

http://www.bjcancer.com

### ALA and ALA hexyl ester-induced porphyrin synthesis in chemically induced skin tumours: the role of different vehicles on improving photosensitization

**A Casas[1], C Perotti[1], H Fukuda[1], L Rogers[2], AR Butler[2] and A Batlle[1]**

[1]Centro de Investigaciones sobre Porfirinas y Porfirias (CIPYP), CONICET and Department of Biochemistry, School of Sciences, University of Buenos Aires, Argentina; and [2]School of Chemistry, University of St Andrews, St Andrews, Fife, UK

**Summary** Exogenous administration of 5-aminolevulinic acid (ALA) is becoming widely used to enhance the endogenous synthesis of Protoporphyrin IX (PpIX) in photodynamic therapy. We analysed porphyrin formation in chemically induced squamous papillomas, after topical application of ALA and ALA hexyl ester (He-ALA) administered in different formulations, as well as the pattern of distribution in the internal organs, and the synthesis of porphyrins in distant tumoural and normal skins. A lotion formulation containing DMSO and ethanol was the best vehicle for topical ALA delivery to papillomas, whereas cream was the most efficient formulation for He-ALA application. Similar porphyrin concentration can be accumulated in the skin tumours employing either ALA or He-ALA delivered in their optimal formulations. The use of cream as a vehicle of both ALA and He-ALA, induces highest porphyrin tumour/normal skin ratios. The main advantage of using He-ALA is that porphyrins synthesized from the ester are more confined to the site of application, thus inducing low porphyrin levels in normal skin, liver, blood and spleen, as well as in papillomas distant from the point of application, independently on the vehicle employed, so reducing potential side effects of photodynamic therapy. © 2001 Cancer Research Campaign http://www.bjcancer.com

**Keywords**: ALA; ALA esters; photodynamic therapy; squamous papillomas

Photodynamic therapy (PDT) is a very promising antineoplastic treatment, specially for superficial skin tumours, based on the preferential accumulation of a photosensitizer in malignant tissue after its administration. Light of an appropriate wavelength illumination excites the photosensitizer and subsequent photoactivation results in the release of cytotoxic substances such as singlet oxygen or free radicals which are responsible for the destruction of PDT treated tissue (Dougherty et al, 1984).

At the beginning of the 1990s 5-aminolevulinic acid (ALA), a precursor of the endogenous photosensitizer protoporphyrin IX (PpIX), began to be used to induce the selective accumulation of tetrapyrroles endogenously synthesized in the tumour tissue (Kennedy and Pottier, 1990; Fukuda et al, 1992).

ALA-mediated photodynamic therapy (ALA-PDT) shows promise in skin precancerous stages such as solar keratoses, Bowen's disease, and tumours including basal cell carcinomas, Paget's disease and squamous cell carcinomas (Kurwa and Barlow, 1999). Nonmalignant skin pathologies such as psoriasis (Boehncke et al, 1994) and actinic keratoses, among others, have also been treated by ALA-PDT (Fritsch et al, 1998).

However, the fact that ALA is a zwitterion at physiological pH and therefore has low lipid solubility, limits its clinical application. ALA poorly passes through biological barriers such as *stratum corneum* of the skin and cellular membranes. Hence, ALA-induced PpIX formation is often restricted to superficial tissue layers because of both inhomogeneous and partial tissue distribution in deeper-lying or nodular lesions (Peng et al, 1995).

It is expected that more lipophilic ALA prodrugs can cross cellular membranes more easily than ALA. After reaching the site of action, the prodrug is enzymatically converted to ALA, which in turn is converted into PpIX. Kloek et al (1996, 1998), Gaullier et al (1997) and Casas et al (2001) found that long chain ALA esters are taken up, hydrolysed to the free acid and transformed into PpIX with higher efficiency than ALA, leading to higher photosensitiser levels both in vivo and in vitro.

Some ALA esters have been used in normal mouse skin (Peng et al, 1996) and in human basal cell carcinomas (Peng et al, 1995), and a higher and more homogeneous tissue distribution was produced when compared to that of free ALA-induced porphyrins.

Van den Akker et al (2000a) using hairless mice showed that ALA pentyl ester produces only slightly more PpIX than ALA in UVB-induced (pre)cancerous skin lesions, while in normal skin, porphyrin levels were equal from both ALA compounds. The same authors (Van den Akker et al, 2000b), found in normal mice skin that ALA hexyl ester was not better than ALA in synthesizing PpIX. In both studies, ALA and ALA esters were delivered in a cream formulation.

Another approach aimed at improving the pro-drug skin penetration is the use of different vehicles and enhancers for their administration (Casas et al, 1999a, 2000a).

The aim of this work was to modulate and optimize porphyrin accumulation and tumoural selectivity, employing different vehicles and the penetration enhancer DMSO, in the delivery of ALA and ALA hexyl ester using a murine skin tumour model.

Due to the scarcity of appropriate animal models to study the effectiveness of ALA-PDT, we employed skin tumours induced in SENCAR mice by two-stage initiation/promotion protocol.

*Received 6 June 2001*
*Revised 17 August 2001*
*Accepted 17 September 2001*

*Correspondence to:* A Batlle, Viamonte 1881 10A, 1056 Buenos Aires, Argentina

Initiation of tumorigenesis by the carcinogen 7,12-dimethylbenz[a]anthracene (DMBA), followed by continuous treatment with phorbol ester promoters, such as 12-o-tetradecanoylphorbol-13-acetate (TPA), results in the formation of premalignant papillomas (Burns et al, 1983).

In this paper we have analysed porphyrin formation in chemically induced squamous papillomas, after topical application of ALA and He-ALA administered in different formulations, as well as the pattern of porphyrin distribution in the internal organs, and the synthesis of porphyrins in distant tumoural and normal skins.

### MATERIALS AND METHODS

### Animals and drugs

Six- to eight week-old female SENCAR mice were provided by the Comisión Nacional de Energía Atómica Argentina (CNEA). Animals were housed separately, acclimatized before use, subjected to a 12 h light/ 12 h dark cycle and fed with mice chow (Molinos Rio de la Plata, Argentina), and water ad libitum.

ALA, 7,12-dimethylbenz[a]anthracene (DMBA) and 12-o-tetradecanoylphorbol-13-acetate (TPA), were purchased from SIGMA Chem Co., St Louis, MO, USA. ALA hexyl-ester (He ALA) was synthesized according to the method previously described by Casas et al (1999b). All other chemicals were of analytical grade.

### Induction of tumours in SENCAR mice skin

Chemical carcinogen-induced benign squamous papillomas were developed employing the following protocol: 6–8-week-old female SENCAR mice were shaved. Only those animals in resting phase of the hair cycle were used in the tumour protocol and treated topically on the dorsal shaved area with a single topical application of DMBA (20 µg in 0.2 ml acetone/mouse). One week later, animals were treated once a week during 4 months with TPA (2 µg in 0.2 ml acetone/mouse) to obtain papillomas. Using this protocol, after 16 weeks, about 50% of the mice treated had an average of three to four tumours per mouse. Few tumours were randomly verified histopatologically as squamous papillomas. Only animals with closely matched tumour size were employed.

Animals were treated in accordance with guidelines established by the Animal Care and Use Committee of the Argentine Association of Specialists in Laboratory Animals (AADEALC), in full accord with the UK Guidelines for the Welfare of Animals in Experimental Neoplasia (UKCCCR, 1988).

### Preparation and administration of ALA formulation

Saline solution: the hydrochloric salt of ALA and He-ALA were dissolved in saline at a concentration of 150 and 225 mg/ml respectively immediately before use. Saline/ethanol: 40% ethanol in saline. Saline/DMSO: 10% dimethylsulfoxide in saline. Saline/DMSO/ethanol: 10% DMSO and 40% ethanol in saline. Cream: ALA and He-ALA were daily prepared at 20% and 30%, respectively, in an oil in water emulsion cream (Genargen, Argentina). A total amount of 15 mg ALA and 22.5 mg He-ALA per mouse was applied in order to obtain an equimolar free ALA concentration for all formulations. In tumour bearing mice, ALA and He-ALA in different formulations were topically applied over a single papilloma surface (weighing approximately 100 mg) plus a 3 mm peritumoural margin rubbing in the surface during a period of 5 min, time at which no vestiges of either cream or lotion are visible. Before any application, mice received mild anaesthesia (Fentanyl and Diazepam). All formulations were applied under occlusive dressing to avoid distribution of the compounds.

For porphyrin determinations in both skin and internal organs, mice having 3–4 papillomas were employed. The peritumoural skin (3 mm margin of skin surrounding the treated papilloma) was carefully removed from the tumour itself. The rest of the non-treated papillomas were pooled for porphyrin extraction. Samples of distant normal skin were excised from the ventral area of the mice.

In experiments aimed at studying porphyrin distribution as a function of the distance of ALA application, 7–8 tumour-bearing mice were employed. In this case, a single papilloma was topically treated as explained above and the distant papillomas were processed separately for porphyrin measurement. In tumour-free mice, ALA or He-ALA was applied and rubbed in over an area of 1.76 $cm^2$ in diameter of normal skin, and another three distant normal skins were excised from different consecutive zones. All the skins were shaved before processing.

Optimal time and precursor concentrations were chosen from previous work (Casas et al, 2000b).

### Tissue porphyrin extraction

After 3 hr of ALA or He-ALA topical application, animals were sacrificed. Before killing, mice were injected with heparin (0.15 ml, 1000 UI) and after sacrifice, they were perfused with 200 ml of sterile saline. The tissue samples were homogenized in a 4:1 solution of ethyl acetate: glacial acetic acid mixture. Blood samples were heparinized and vigorously vortexed with the same extraction solvents. The mixtures were centrifuged for 30 min at 3000 ***g***, and the supernatants were added with an equal volume of 5% HCl. Extraction with HCl was repeated until there was no detectable fluorescence in the organic layer. The aqueous fraction was used for the determination of porphyrins. For fluorometric determination, a Shimadzu RF-510 spectrofluorometer was used, with an emission wavelength of 604 nm and an excitation wave length of 406 nm, employing a PpIX reference standard.

### Statistical analysis

The unpaired *t*-test was used to establish the significance of differences between groups. Differences were considered statistically significant when $P < 0.05$. Three mice per group were employed.

### RESULTS

Figures 1 and 2 show porphyrin synthesis in treated papillomas, distant papillomas, normal skin surrounding papillomas and normal distant skin after topical application of ALA and He-ALA respectively, applied in different formulations. The cream vehicle is twice as efficient as saline lotion at inducing porphyrin synthesis in papillomas treated with ALA (Figure 1). However, by adding ethanol, or DMSO and ethanol to the saline formulation, but not DMSO alone, tetrapyrrole accumulation can be significatively increased when compared with saline lotion ($P = 0.001$ and $P = 0.0007$ respectively). Saline/DMSO/ethanol vehicle induces 3.5 times more porphyrin accumulation than saline alone. A completely different pattern is observed in papillomas treated with He-ALA

*© 2001 Cancer Research Campaign*

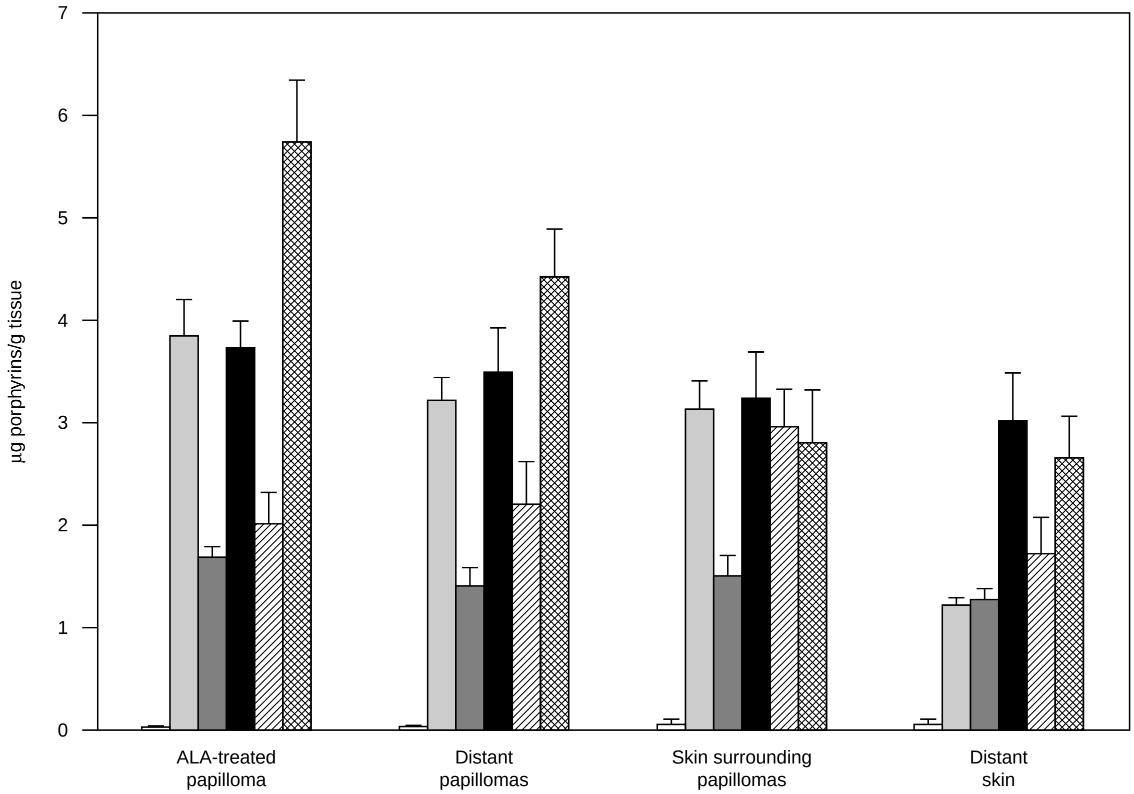

**Figure 1** Porphyrin synthesis in skin of tumour-bearing mice after topical application of ALA. Porphyrins were determined 3 h after ALA application in different vehicles: (□) control without any treatment, cream (□), saline lotion (■), saline/ethanol (■), saline/DMSO (▨) and saline/DMSO/ethanol (▩). Bars represent porphyrin values (mean ± SD)

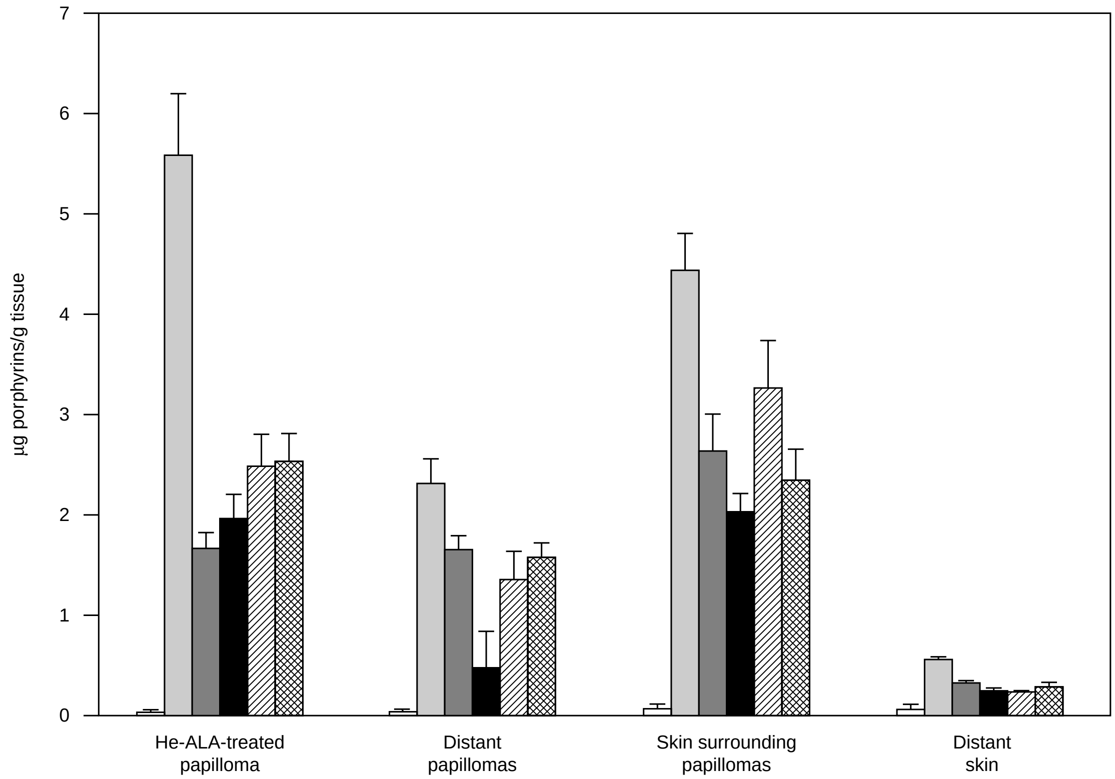

**Figure 2** Porphyrin synthesis in skin of tumour-bearing mice after topical application of He-ALA. Porphyrins were determined 3 h after He-ALA application in different vehicles: (□) control without any treatment, cream (□), saline lotion (■), saline/ethanol (■), saline/DMSO (▨) and saline/DMSO/ethanol (▩). Bars represent porphyrin values (mean ± SD)

 *© 2001 Cancer Research Campaign*

(Figure 2), where cream is the best vehicle, inducing 3.5 times more porphyrins than saline lotion; addition of DMSO to saline He-ALA can increase porphyrin synthesis by a factor of 1.5.

The amount of porphyrins in distant papillomas is similar to that found in ALA-applied papillomas, independently on the vehicle, except for the saline/DMSO/ethanol formulation, inducing a significant lower tetrapyrrole accumulation in the distant tumour ($P = 0.03$). When using He-ALA formulations, porphyrin concentrations measured in distant papillomas, except for saline lotion, in all other vehicles are half the amount synthesized in He-ALA treated papillomas.

In general, peritumoural skins surrounding papillomas, which were also treated with ALA or the ALA ester, exhibit porphyrin levels similar to those found in ALA and He-ALA treated papillomas. The notorious exception is the peritumoural skin treated with ALA in saline/ethanol/DMSO, which accumulated half the amount of tumoural porphyrins.

In distant normal skin tissue of mice treated with ALA or He-ALA, porphyrin levels are lower than those of treated tumors, but treated papilloma to distant normal skin ratios depend on the vehicle employed, and in general they are much higher for He-ALA formulations (Table 1). The use of cream as a vehicle for both ALA and He-ALA, induces higher ratios. However, in the case of He-ALA, but not ALA, when either ethanol or DMSO are added to the saline formulation, the ratios are largely increased.

Figures 3 and 4 show porphyrin accumulation in internal organs after topical application of papillomas with ALA and He-ALA respectively. As a general pattern for all vehicles, porphyrin accumulation in liver, kidney, spleen and blood is higher in tumour-bearing mice topically treated with ALA as compared with the He-ALA-treated mice, and the factor of increase depends on the tissue.

**Table 1** Papilloma to normal distant skin porphyrin ratios. Porphyrins were determined 3 h after application of ALA or He-ALA to a single papilloma in different vehicles. Papilloma/ normal skin porphyrin ratios were calculated with data from Figures 1 and 2

| | ALA ratio | He-ALA ratio |
|---|---|---|
| Control[a] | 0.6 | 0.6 |
| Cream | 3.2 | 9.6 |
| Saline lotion | 1.3 | 4.9 |
| Saline/ethanol | 1.2 | 7.2 |
| Saline/DMSO | 1.1 | 9.9 |
| Saline/DMSO/ethanol | 2.1 | 8.1 |

In Figure 5 we demonstrate that porphyrins formed from ALA saline lotion in normal skin of a non-tumour bearing mice, do not depend on the distance from the point of application, and there is no selectivity for the site of application. The use of the cream vehicle for ALA application induces a more confined porphyrin accumulation in the site of application, although in the ventral side of the animal (75 mm distance), porphyrin concentration is not different from that formed in the treated zone. In He-ALA-treated skin, both saline and cream induce an accumulation of porphyrins strongly dependent on the distance from the site of application, and it is the cream vehicle the one inducing lower porphyrin accumulation in distant skins.

In Figure 6 it is shown that after application of ALA lotion to a single papilloma, porphyrins are uniformly distributed to all distant papillomas, whereas He-ALA induces a higher concentration in the

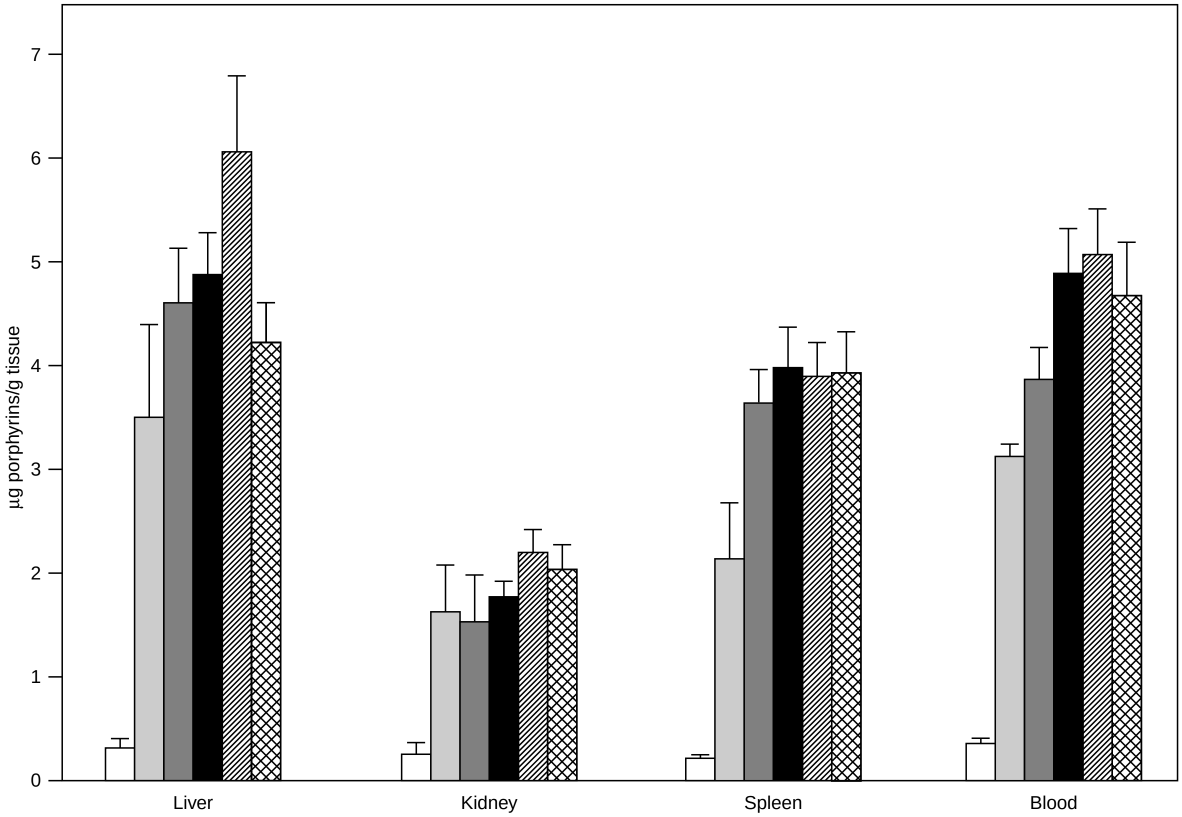

**Figure 3** Porphyrin synthesis in tissues of tumour-bearing mice after topical application of ALA. Porphyrins were determined 3 h after ALA application in different vehicles: (□) control without any treatment, cream (□), saline lotion (■), saline/ethanol (■), saline/DMSO (▨) and saline/DMSO/ethanol (☒). Bars represent porphyrin values (mean ± SD) expressed in µg/g tissue and µg/ml blood

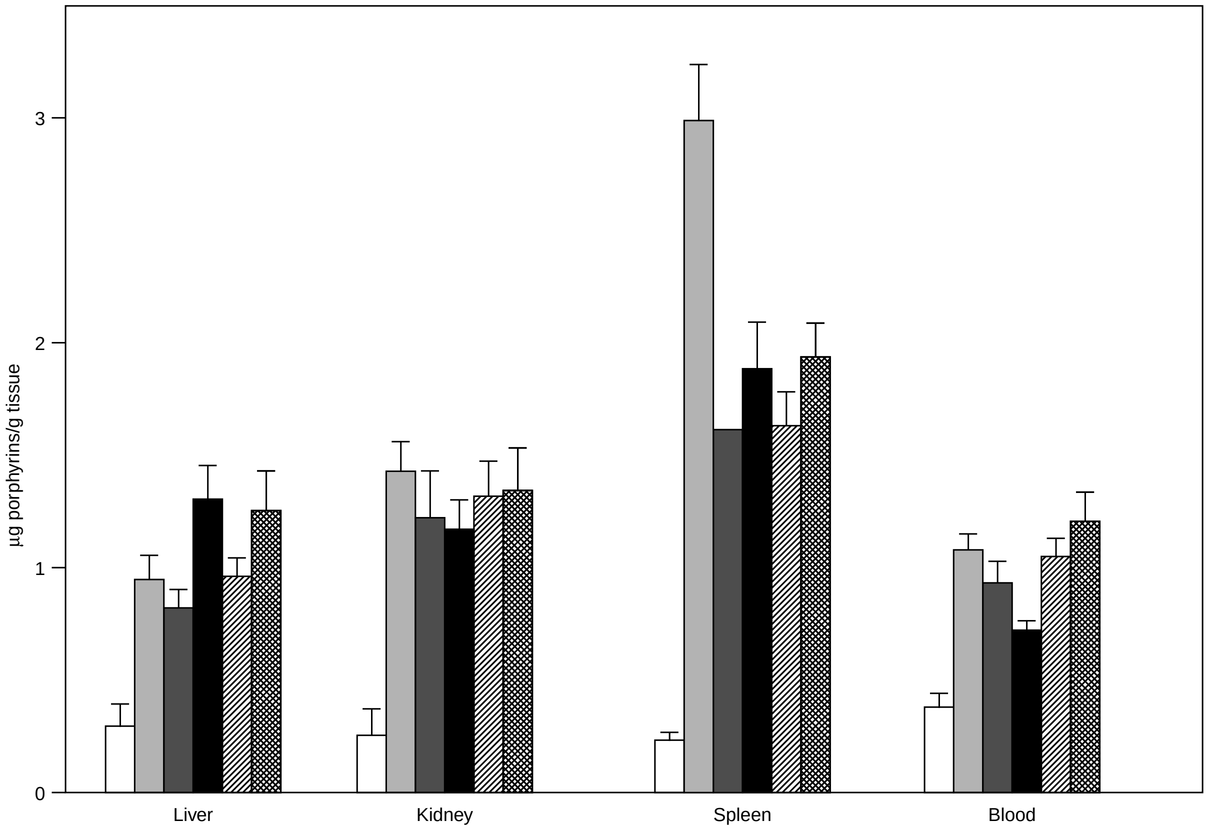

**Figure 4** Porphyrin synthesis in tissues of tumour-bearing mice after topical application of He-ALA. Porphyrins were determined 3 h after He-ALA application in different vehicles: (□) control without any treatment, cream (□), saline lotion (■), saline/ethanol (■), saline/DMSO (▨), and saline/DMSO/ethanol (▩). Bars represent porphyrin values (mean ± SD) expressed in µg/g tissue and µg/ml blood

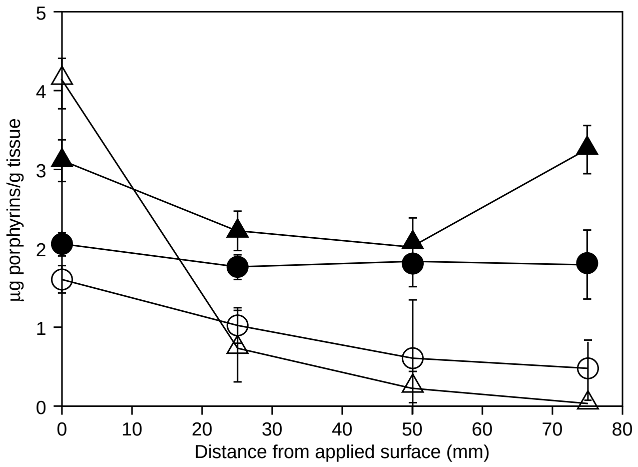

**Figure 5** Distribution pattern of normal skin porphyrins from non-tumour bearing mice after ALA and He-ALA application in a normal skin area. Porphyrins were measured 3 h after application of ALA saline lotion (●), He-ALA saline lotion (○), ALA cream (▲) He-ALA cream (△)

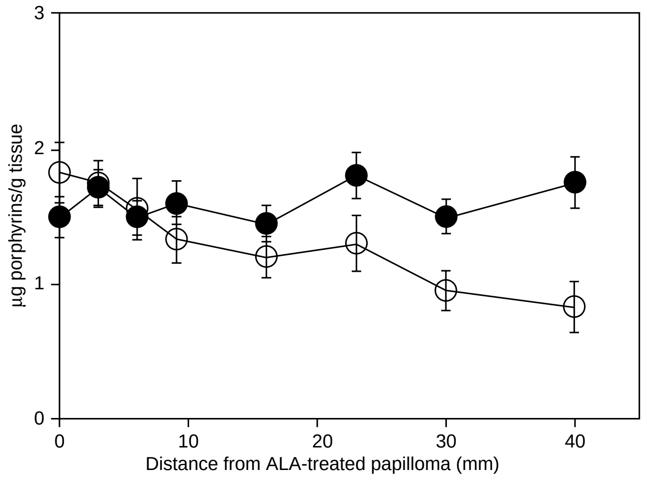

**Figure 6** Distribution pattern of papilloma porphyrins from tumour bearing mice after ALA and He-ALA application in a single papilloma. Porphyrins were measured 3 h after ALA (●) or He-ALA (○) saline lotion topical application treated papilloma and porphyrin accumulation in the non-treated tumours decreases with the distance from the He-ALA applied papilloma.

### DISCUSSION

From these results, we can conclude that similar porphyrin concentrations can be reached in the skin tumours employing either ALA or He-ALA delivered in their optimal formulations employing equimolar amounts of the prodrugs. A lotion formulation containing DMSO and ethanol is the best vehicle for topical ALA delivery to papillomas, whereas cream is the most efficient formulation for He-ALA application. However, the use of cream as a vehicle of both ALA and He-ALA, induces the highest porphyrin tumour/normal skin ratios. As a consequence, the cream vehicle should be the election for the treatment of skin

*© 2001 Cancer Research Campaign*

malignancies with He-ALA. In the case of ALA, although saline/ethanol/DMSO induces higher porphyrin tumour levels, the use of cream should also be recommended when higher selectivity is needed.

Some vehicles induce differential porphyrin accumulation. For example, saline/ethanol/DMSO as the ALA vehicle induces a surprisingly low porphyrin accumulation in distant papillomas as well as in peritumoural skin as compared to porphyrin levels accumulated in the treated tumours. In addition, He-ALA applied as saline lotion induces higher porphyrin values in distant papillomas when comparing with the treated to distant papillomas ratios for the other vehicles.

In general, a similar amount of porphyrins is synthesized in distant papillomas from ALA when compared with ALA-applied papillomas, demonstrating that either ALA itself and/or the porphyrins formed by synthesis can be distributed throughout the vasculature and accumulate in distant tumours. On the other hand, lower porphyrin synthesis occurs in distant papillomas from He-ALA treated papillomas, showing that He-ALA and/or porphyrins formed from the precursor, are more selectively localized in the site of application.

Looking at tissues far from the site of application, distant normal skin porphyrin levels are lower than porphyrin levels in distant papilloma tissues, showing that porphyrins accumulate preferentially in tumour than in normal skin, even though either ALA or He-ALA were applied in remote zones.

Peritumoural skins surrounding papillomas, which were also treated with the porphyrin precursors, exhibit porphyrin levels similar to those found in both ALA and He-ALA treated papillomas. This demonstrates that when either ALA or its derivative is applied on tumoural or normal skin, the induced porphyrin synthesis is the same, and that special care must be taken to restraining ALA application to the affected skin.

In addition to the selectivity of prophyrin accumulation from He-ALA showed in skin tissues, the amount of tetrapyrroles accumulated in internal organs is much lower in mice treated with He-ALA than in those treated with ALA. Even comparing ALA in saline/DMSO/ethanol with He-ALA in cream, the best vehicle for each compound, porphyrin synthesis in liver and blood is four times lower in the former demonstrating that, independently on the vehicle, the use of ALA hexyl ester provides a clear advantage in selective porphyrin accumulation.

Peng et al (1996) applied ALA and other ALA esters (methyl, ethyl and propyl) to normal mouse skin and detected tissue fluorescence by means of an optical-based point monitoring system in situ. They found no porphyrins in areas other than that in which the ALA ester cream was applied, while in the case of ALA, a significant fluorescence was seen in the skin outside the application area. In our work, employing porphyrin extraction procedures, fluorescence values in distant skins as well as in internal organs were also much lower for He-ALA than ALA-treated mice, although in both cases they were much higher than basal levels.

The confinement to the site of application achieved by the use of ALA hexyl ester demonstrates that ALA, and not porphyrin, is the main molecule likely to be distributed through the bloodstream. Topically applied He-ALA may be retained by *stratum corneum*, which may act as a reservoir (Rougier and Lotte, 1986), and diffuse superficially, leading to the pattern of skin porphyrin accumulation dependant on the distance of application.

In this regard, Van den Akker et al (2000b) demonstrated that the *stratum corneum* was the main barrier for He-ALA penetration. They found that the ester of ALA did not induce higher PpIX production in normal mouse skin compared to ALA. Employing a cream formulation and tape stripping of the *stratum corneum* they demonstrated that this was the layer limiting and lowering PpIX production.

The same group (Van den Akker et al, 2000a) employed a shorter ester of ALA, the ALA pentyl ester, and found higher PpIX levels in the *stratum corneum* of lesional UVB-exposed mouse skin, but not in the dysplastic layer of the epidermis, showing that this ester also poorly diffusses as compared with ALA.

In our work, porphyrin accumulation in internal organs after topical He-ALA application to papillomas, shows that the hexyl ester of ALA does reach the dermis. Moreover, after applying He-ALA in normal skin of non-tumour bearing mice, we also found the same pattern formed in internal organs for porphyrin accumulation. Our procedure of applying the creams and lotions by rubbing in the surface during 5 min, both in lesional and normal skin, may be facilitating the passage of the ALA ester to deeper layers. Different procedures of ALA application avoiding the rubbing in of the creams and lotions are currently under investigation.

An additional explanation to the confinement of He-ALA is that the intracellular conversion of the ester to free ALA in the actual site of application may be preventing the efflux of the hydrolysed ALA to the bloodstream to reach non-tumour tissues.

The confinement of porphyrins and, very likely of the He-ALA, implies the avoidance of potential unwanted side effects. In the case of topical use of ALA to discrete lesions, although skin photosensitivity and liver porphyrin accumulation have not proved to be a clinical problem, the confinement of He-ALA itself to the site of application would be preventing any possible normal tissue cytotoxicity. Although the toxicological profile of He-ALA is poorly characterized at present, we have reported dark cytotoxicity after exposure of a tumour cell line to concentrations of He-ALA as high as 1.4 mM (Casas et al, 2001). Moreover, another future clinical applications of ALA-PDT requiring larger amounts of pro-photosensitizer, such as the treatment of early stage cutaneous T-cell lymphomas and breast secondaries in the chest wall would also be expected to profit from the benefits of porphyrin restraint.

We conclude that, by varying the vehicle composition it is possible to obtain similar porphyrin levels employing either ALA or its hexyl ester derivative. We wish to emphasize that He-ALA has the additional advantage of confining porphyrin synthesis to the site of its topical application, either on the tumour or on normal skin, more efficiently than ALA.

### ACKNOWLEDGEMENTS

This research was supported by grants from the Argentine National Research Council (CONICET) (PIP 4108/96 and 105508/98–99), the Science and Technology Argentine Agency (STAA) (PICT 05–00000–01861) and the Association for International Cancer Research (AICR-UK, 98–6). The authors are very grateful to Mrs Victoria Castillo for her skillful technical assistance, and to Dr Hebe Duran and Beatriz Molinari del Rey for the initiation step in chemical tumour induction and animal provision. AM del CB and HF hold the posts of Superior and Associate Researchers at the CONICET. CP is a 'Carrillo-Oñativia' fellow, Ministerio de Salud Pública.

*© 2001 Cancer Research Campaign*

### REFERENCES

Boehncke W, Sterry W and Kaufmann R (1994) Treatment of psoriasis by topical photodynamic therapy with polychromatic light. *Lancet* **343**: 801

Burns F, Albert R, Altshuler B and Morris E (1983) Approach to risk assessment for genotoxic carcinogens based on data from the mouse skin initiation-promotion model. *Environ Health Perspect* **50**: 309–320

Casas A, Fukuda H and Batlle A (1999a) Tissue distribution and kinetics of endogenous porphyrins synthesized after topical application of ALA in different vehicles. *Br J Cancer* **81**: 13–18

Casas A, Batlle A, Butler A, Robertson D, Brown E, MacRobert A and Riley P (1999b) Comparative effect of ALA derivatives on Protoporphyrin IX production in human and rat skin organ cultures. *Br J Cancer* **80**: 1525–1532

Casas A, Fukuda H, Di Venosa G and Batlle A (2000a) The influence of the vehicle on the synthesis of porphyrins after topical application of 5-aminolevulinic acid. Implications in cutaneous photodynamic sensitization. *Br J Dermatol* **143**: 564–572

Casas A, DiVenosa G, Casentini C, Meiss R, Vanzulli S, Fukuda H and Batlle A (2000b) Terapia fotodinámica en el tratamiento de tumores de piel inducidos quámicamente. *Rev Argent Dermatol* **81**: 144–152

Casas A, Fukuda H, Di Venosa G and Batlle A (2001) Photosensitisation and mechanism of cytotoxicity induced by the use of ALA derivatives in photodynamic therapy. *Br J Cancer* (In press)

Dougherty T (1984) Photodynamic therapy (PDT) of malignant tumors. *Crit Rev Oncol Hematol* **2**: 83–116

Fritsch C, Lang K, Neuse W, Ruzicka T and Lehmann P (1998) Photodynamic diagnosis and therapy in dermatology. *Skin Pharmacol Appl Skin Physiol* **11**: 358–373

Fukuda H, Paredes S and Batlle A (1992) Tumor-localizing properties of porphyrins. *In vivo* studies using free and liposome encapsulated aminolevulinic acid. *Comp Biochem Physiol* **102B**: 433–436

Gaullier J, Berg C, Peng Q, Anholt H, Selbo P, Ma L and Moan J (1997) Use of 5-aminolevulinic acid esters to improve photodynamic therapy on cells in culture. *Cancer Res* **57**: 1481–1486

Kennedy J and Pottier R (1990) Photodynamic therapy with endogenous protoporphyrin IX: basic principles and present clinical experience. *J Photochem Photobiol B* **6**: 143–148

Kloek J and Beijersbergen van Henegouwen G (1996) Prodrugs for 5-Aminolevulinic acid for Photodynamic therapy. *Photochem Photobiol* **64**: 994–1000

Kloek J, Akkermans W and Beijersbergen van Henegouwen G (1998) Derivatives of 5-Aminolevulinic acid for Photodynamic therapy: enzymatic conversion into protoporphyrin. *Photochem Photobiol* **67**: 150–154

Kurwa H and Barlow R (1999) The role of photodynamic therapy in dermatology. *Clin Exp Dermatol* **24**: 143–148

Peng Q, Warloe T, Moan J, Heyerdahl H, Steen H, Giercksky K and Nestland J (1995) Distribution of 5-aminolevulinic acid-induced porphyrins in noduloulcerative basal cell carcinoma. *Photochem Photobiol* **62**: 906–913

Peng Q, Moan J, Warloe T, Iani V, Steen H, Bjørseth A and Nesland J (1996) Build-up of esterified aminolevulinic-acid-derivative-induced porphyrin fluorescence in normal mouse skin. *J Photochem Photobiol B* **34**: 95–96

Rougier A and Lotte C (1986) Correlations between horny layer concentration and percutaneous absorption. In: *Pharmacology and skin: 1. Skin Pharmacokinetics*. Shroot & Schaeger: Switzerland

UK-Coordinating Committee on Cancer Research (1988) UKCCCR Guidelines for the Welfare of Animals in Experimental Neoplasia (London, UKCCCR)

Van den Akker J, de Brujin H, Beijersbergen van Henegouwen G, Star W and Sterenborg H (2000a) Protoporphyrin IX fluorescence kinetics and localization after topical application of ALA pentyl ester and ALA on hairless mouse skin with UVB-induced early skin cancer. *Photochem Photobiol* **72**: 399–406

Van den Akker J, Iani V, Star W, Sterenborg H and Moan J (2000b) Topical application of 5-aminolevulinic acid hexyl ester and 5-aminolevulinic to normal nude mouse skin: differences in Protoporphyrin IX fluorescence kinetics and the role of the *stratum corneum*. *Photochem Photobiol* **72**: 681–689

*© 2001 Cancer Research Campaign*
